# Portrait of Dysferlinopathy: Diagnosis and Development of Therapy

**DOI:** 10.3390/jcm12186011

**Published:** 2023-09-16

**Authors:** Camille Bouchard, Jacques P. Tremblay

**Affiliations:** 1 Département de Médecine Moléculaire, Université Laval, Québec, QC G1V 0A6, Canada; camille.bouchard@crchudequebec.ulaval.ca; 2 Centre de Recherche du Centre Hospitalier Universitaire de Québec, Québec, QC G1E 6W2, Canada

**Keywords:** dysferlinopathy, LGMD2B, LGMDR2, Miyoshi myopathy, dysferlin, therapy

## Abstract

Dysferlinopathy is a disease caused by a dysferlin deficiency due to mutations in the *DYSF* gene. Dysferlin is a membrane protein in the sarcolemma and is involved in different functions, such as membrane repair and vesicle fusion, T-tubule development and maintenance, Ca^2+^ signalling, and the regulation of various molecules. Miyoshi Myopathy type 1 (MMD1) and Limb–Girdle Muscular Dystrophy 2B/R2 (LGMD2B/LGMDR2) are two possible clinical presentations, yet the same mutations can cause both presentations in the same family. They are therefore grouped under the name dysferlinopathy. Onset is typically during the teenage years or young adulthood and is characterized by a loss of Achilles tendon reflexes and difficulty in standing on tiptoes or climbing stairs, followed by a slow progressive loss of strength in limb muscles. The MRI pattern of patient muscles and their biopsies show various fibre sizes, necrotic and regenerative fibres, and fat and connective tissue accumulation. Recent tools were developed for diagnosis and research, especially to evaluate the evolution of the patient condition and to prevent misdiagnosis caused by similarities with polymyositis and Charcot–Marie–Tooth disease. The specific characteristic of dysferlinopathy is dysferlin deficiency. Recently, mouse models with patient mutations were developed to study genetic approaches to treat dysferlinopathy. The research fields for dysferlinopathy therapy include symptomatic treatments, as well as antisense-mediated exon skipping, myoblast transplantation, and gene editing.

## 1. Introduction

**Figure 1:**
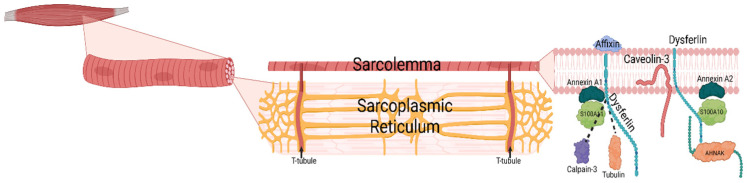
Dysferlin in the sarcolemma. A skeletal muscle is made of fibres. Each fibre is wrapped in a plasma membrane, the sarcolemma, which extends into T-tubules through the sarcoplasmic reticulum. The sarcoplasmic reticulum is made of cavities to stock calcium in the muscles. The sarcolemma is a membrane containing several proteins playing different roles in calcium signalization and membrane repair. Affixin binds to the C-terminal region of dysferlin and shows abnormal localization in dysferlin-deficient muscles [7]. Caveolin-3 also colocalizes with dysferlin, as well as with the dihydropyridine receptor, an L-type Ca^2+^ channel in the T-tubules [8]. Calpain-3 cleaves annexins A1 and A2, as well as AHNAK, to regulate the dysferlin complex and is Ca^2+^-dependant [9]. S100A10 forms a complex with annexin A2 and the C-terminal region of AHNAK and then binds to the dysferlin membrane complex [10]. Annexin A1 also creates a complex with protein S100A11 to join dysferlin in participating in Ca^2+^-dependant membrane repair [11]. Dysferlin also binds with tubulin with its C2A and C2B domains [12].

Dysferlin is a 237 kDa membrane protein located in the plasma membrane [1] and in the transverse tubules of skeletal and cardiac muscles, which are extensions of the sarcolemma inside the muscle fibres [2,3]. The gene encoding for the dysferlin protein is *DYSF* [4], a 55-exon gene [5] on the chromosome 2p13 [6]. It is expressed in various tissues but mostly in skeletal and cardiac muscles, yet it only causes a phenotype in skeletal muscles. Figure 1 indicates the location of dysferlin in the sarcolemma, a membrane on the surface of skeletal and cardiac muscle fibres. Dysferlin is a transmembrane protein, interacting with several other proteins, which have different functions.

Dysferlin has multiple functions, including stabilizing Ca^2+^ signalization and repairing the sarcolemma in skeletal muscles [3,13]. Dysferlin is a part of the ferlin family proteins. They are single-pass membrane proteins with a short C-terminal extracellular domain and multiple C2 cytosolic domains. The C2 domains have different functions in membrane repair, but they are mostly used to reseal the sarcolemma [13]. After muscular effort, the plasma membrane is damaged, and the lesions let Ca^2+^ enter the cell and recruit vesicles to seal the lesions [14].

## 2. The Roles of Dysferlin

Dysferlin seems to play more than one role. In fact, it takes part in membrane repair and vesicle fusion, in T-tubule development and maintenance, in Ca^2+^ signalling, and in phagocytosis [15]. It also regulates myogenin, as well as decay-accelerating-factor DAF/CD55 in skeletal muscles [16].

More specifically, dysferlin is involved in membrane repair and vesicle fusion with its seven C2 calcium-binding domains, which are known for calcium-dependant activities, such as membrane fusion and repair [17]. A V67D point mutation in the C2A domain of dysferlin impairs its ability to bind phospholipid [18]. To repair a damaged membrane, vesicles bring membrane components from intracellular sources and fuse with the membrane at the lesion site [19]. Kobayashi et al. [17] investigated the membrane repair process in vitro, and their model suggests that physical activity leading to lesions in the membrane is followed by the influx of calcium through the membrane. Kinesin and myosin interact with mitsugumin 53 (MG53) to deliver intracellular vesicles to the damaged site, which fuse to the plasma membrane, using dysferlin and annexins A1 and A2 to seal the lesion.

The role of dysferlin in T-tubule development has also been studied. Hofhuis et al. showed that dysferlin is responsible for T-tubule biogenesis and that C2 domains are necessary for this function, as truncated forms of dysferlin do not lead to tubule formation [20]. This study as well as Therrien et al. showed that dysferlin binds to phosphatidylinositol 4-phosphate and phosphatidylinositol 4,5-bisphosphate (PI(4,5)P2), a phospholipid, and recruits them to generate T-tubules [21].

Dysferlin also mediates Ca^2+^ signalling by stabilizing stress-induced Ca^2+^ homeostasis in T-tubule membranes, near the triad junction. In fact, Muriel et al. studied the role of each dysferlin C2 domain [13]. Their conclusion was that some domains are responsible for Ca^2+^ signalling and others play a role in the membrane repair process independently. They also noticed that the absence of dysferlin leads to a decreased amplitude of voltage-induced Ca^2+^ transients, and they formulated the hypothesis that dysferlin plays a role at the triad junction in optimizing the interaction between the L-type Ca^2+^ channel (LTCC) and the ryanodine receptor (RyR1). Wang et al. showed that the dysferlin C2A domain binds with two calcium ions to have a more rigid structure and promote more calcium binding [22].

A lack of dysferlin, along with mutations in the *DYSF* gene, results in aberrant phagocytosis. In fact, patients and DYSF-deficient mouse monocytes have increased phagocytosis activity [23]. Nagaraju et al. knocked down *DYSF* mRNA and noticed significantly enhanced phagocytosis activity. Therefore, their conclusion was that the increased phagocytosis activity was caused by the dysferlin deficiency rather than by the muscle degeneration. The hypothesis is that dysferlin-deficient damaged myofibers cause an inflammation response leading to a cascade increasing the expression of small Rho family GTPases RhoA, Rac1, and Cdc 42 [23].

Dysferlin also regulates other factors such as myogenin and decay-accelerating factor DAF/CD55 since they are both downregulated in dysferlin-deficient muscles [16]. DAF/CD55 is only downregulated in the skeletal muscles of dysferlin-deficient patients but not in the cardiac muscle, which could explain the prevalence of the phenotype affecting skeletal muscles. However, it remains unclear which functional deficit leads to the manifestation of dysferlinopathy. For example, restoring membrane repair does not reverse all aspects of the disease [24].

## 3. Dysferlinopathy

In the ClinVar database, there are 719 hereditary mutations in the *DYSF* gene that have been classified as either pathogenic or likely pathogenic [25] and that can cause a dysferlinopathy, such as Miyoshi Myopathy type 1 (MMD1) and Limb–Girdle Muscular Dystrophy type 2B (LGMD2B), now called Limb–Girdle Muscular Dystrophy R2 dysferlin-related (LGMDR2) [4,26]. LGMDR2 first affects proximal muscles (such as thighs), and MMD1 affects distal muscles initially (such as calves) [27]. The same mutations in the *DYSF* gene can cause either an MMD1 or an LGMDR2 presentation, leading to family members with the same mutation being affected in different muscles [28,29]. Since both clinical presentations can be acquired by the same mutations and progress the same, the term dysferlinopathy is used, as it is the same disease [30].

### 3.1. Signs and Symptoms

The age of onset for MMD1 and LGMDR2 is 20 ± 5 years and 21 ± 7 years, respectively, and patients present calf or thigh weakness and atrophy. In the following 5 years, the weakness and atrophy evolve to include the upper limbs [31]. Most patients (72%) present with lower limb weakness, which is proximal for 15%, distal for 32%, or both for 25% of patients. An upper limb weakness is also reported by 7% of patients. Their symptoms include muscle wasting (27%); pain, stiffness, or cramps (13%); or pseudohypertrophy (6%) [32]. Patients’ creatine kinase (CK) level is also on average 54 times higher than that of control groups [31], and they start using a cane to walk in their thirties and become wheelchair-bound in their forties or, on average, 21 years after onset [33]. The phenotype is located in the skeletal muscles, and the heart remains unaffected, with normal results for electrocardiogram and echocardiogram [34]. However, a 3-year study showed that up to 30% of patients have a reduced forced vital capacity (FVC) and that up to 58% of patients have a cardiac P-wave abnormality, which can be a risk factor for atrial flutter [35].

### 3.2. MRI Pattern

A minority of patients do not correlate with the typical phenotype, but it has been described as follows in Table 1 [36]:

For dysferlinopathy, there is no significant difference between the right and left muscle fat fraction (FF) or the contractile cross-section area (cCSA); it is therefore considered symmetrical [37]. However, FF increases significantly with time after the onset and even more so in non-ambulant patients. The fastest increases in FF occur in the quadriceps, hamstrings, adductors, and posterior leg muscles. cCSA also decreases concomitantly with time. A three-year study concluded in 11.0% and 12.8% increases in cCSA, while 9.6% and 8.4% increases in FF were observed in ambulant and non-ambulant patients compared to the control group [37].

Another MRI study revealed that the most frequently affected muscles were the gastrocnemius medialis and soleus, with a similar pattern for both dysferlinopathy phenotypes [38]. It also confirmed the correlation between time since onset and the severity of muscle pathology. This study found asymmetry in 41.8% of patients. The study pointed out two commonly affected muscles in the arms, namely, the biceps brachii (for 57.1% of patients) and the forearm anterior muscles (for 53.8% of patients), and several affected muscles in the scapular girdle, namely, the subscapularis (80.8%), latissimus dorsi (75.3%), infraspinatus (73.8%), and supraspinatus (72.8%). As for the pelvic girdle and trunk region, the most affected muscles were the tensor fascia latae (95%), gluteus minimus (90.8%), obturator externus (86%), iliocostalis (93.1%), longissimus (86.2%), and multifidus (88.5%). The most commonly involved thigh muscles were the semimembranosus (95.4%), semitendinosus (90.2%), biceps femoris long head (93.5%), and adductor magnus (94.1%). In the lower leg, the affected muscles were the soleus (99.45%), gastrocnemius medialis (99.45%), and gastrocnemius lateralis (94.7%). The study concluded that spinal muscles were equally or more affected than abdominal ones and that the anterior muscles in the forearm were equally or more affected than the posterior ones. They also observed that all symptomatic patients had at least one affected posterior lower leg muscle and that severely affected patients had involvement of all lower leg muscles.

### 3.3. Muscle Biopsy

Patient muscle biopsy shows variability in muscle fibre sizes. Some fibres are necrotic, and others are regenerative [39]. Microscopic analyses show proliferating connective tissue. Other studies confirmed the abnormal variability in the size of fibres along with splitting fibres and scattered necrotic and regenerating fibres [40]. They also noticed a high quantity of internalized nuclei, as well as increased endomysial and perimysial connective tissue. They also found granular membrane attack complex (MAC) deposits on the surface of non-necrotic fibres, which was also described in previous studies [40,41]. An electron microscopy analysis of biopsies showed small defects of the plasma membrane, especially in hypercontracted or necrotic fibres. Small vesicles also formed layers to replace the sarcolemma on the surface of the muscle fibres. This study also showed the thickening or duplication of the basal lamina in 35% of dysferlin-deficient patient fibres compared to a control group. Their fibres also showed papillary projections surrounded by globular dense material. Their subsarcolemmal region also contained small vacuoles and an increase in rough endoplasmic reticulum.

Several other studies confirmed that typical patient biopsies contain necrotic fibres, regenerative ones, and connective or fat tissue [42,43,44]. One of them compared the phenotypes of early-onset (EO) and late-onset (LO) patients. Their results showed a non-significant trend for EO patients to have more perimysial inflammation, necrotic fibres, and fat and connective tissue accumulation [42]. Some results came from case studies and vary from patient to patient. DeLuna et al. also showed that dysferlin is upregulated in the activated satellite cells of dysferlinopathy patients, especially during differentiation into myotubes [45].

### 3.4. Diagnosis

Dysferlinopathy has common symptoms with other diseases such as polymyositis (PM) or other Limb–Girdle muscular dystrophies (LGMDs), and it can therefore be misdiagnosed [46]. In fact, they all present with a high creatine kinase level, and muscle inflammation and weakness.

Another possible but rare misdiagnosis for dysferlinopathy can be Charcot–Marie–Tooth disease (CMT), since both diseases involve a distal weakness phenotype [34]. One way to tell them apart is that CMT does not cause a high creatine kinase level nor the sarcolemma upregulation of major histocompatibility complex class I (MHC I) like dysferlinopathy does. An electromyographic (EMG) study makes it possible to differentiate dysferlinopathy from CMT [47].

A Western blot can show whether a patient has an absence of the dysferlin protein, but it cannot confirm a mutation. Genetic screening is necessary to confirm whether their *DYSF* gene contains two pathogenic or potentially pathogenic mutations. This can be carried out on either a blood or muscle sample [48,49,50]. More precisely, a Western blot with a dysferlin-targeting antibody can verify the presence or absence of the dysferlin protein in the tissues, such as a muscle biopsy, but this can also be a secondary decrease due to another protein, such as calpain-3 [51]. Direct sequencing or different hybridization methods can allow one to identify precise mutations in the gene [52].

### 3.5. Clinical Research

The evolution of 193 patients with dysferlinopathy over one year was evaluated at baseline, 6 months, and one year with tests such as the ACTIVLIM questionnaire, an adapted North Star Ambulatory Assessment (a-NSAA), the Motor Function Measure (MFM-20), timed function tests, the 6-minute walk test (6MWT), the Brooke scale, the Jebsen test, manual muscle testing, and hand-held dynamometry [53]. The conclusion was that it is possible to measure changes in dysferlinopathy patients within 6 months using a-NSAA, MFM-20, a timed 10 m walk, and timed up and go.

Also, a study was conducted to evaluate the impact of dysferlinopathy on patients’ function and quality of life [54]. This study could be used to assess the right tests to quantify patient needs in future research for a cure or for services to help patients improve their quality of life and daily functions.

## 4. Mouse Models

There are different dysferlin-deficient mouse models available for research. Different methods are used to create these models. Some consist in suppressing one or more exons or inserting a retrotransposon into an intron. Recent models also carry a point mutation from patients, and others contain a partial or complete human dysferlin transgene.

The **BlaJ mouse model** is an A/J mouse on a black 6 (B6) background and is the most frequently used mouse model to study dysferlinopathy [55,56]. These mice show the first dystrophic signs at 2 months, which are centronucleated fibres and inflammation. Muscular impairment is seen in the majority of muscles, especially in the *psoas*, *quadriceps femoris*, and *TA*. It has the advantage of being less sensitive to infections than the A/J mouse model [55].

The **A/J mouse model** contains a 6000 bp retrotransposon that spontaneously inserts itself into intron 4 of dysferlin, causing the disruption of the splicing of the *DYSF* gene and absent dysferlin protein expression. It has a comparable phenotype to Dysf −/− yet shows later onset at 4–5 months [57]. It also shows abdominal and proximal muscle deterioration, with only a mild distal phenotype (https://www.jax.org/strain/000646 (accessed on 1 August 2023)).

**Dysf −/− homozygous** mice have a neomycin-resistant gene replacing the last three exons of *DYSF*, coding for the transmembrane domain. These mice show the first pathological symptoms at 2 months [57], characterized by degenerating and regenerating muscle fibres and central nuclei. The first affected muscles are proximal muscles (quadriceps femoris and triceps brachii) as opposed to distal muscles (gastrocnemius, soleus, and tibialis anterior), which do not show a pathological phenotype before 5 to 6 months. The abdominal muscles also become affected at 6 months. These mice also gradually present human pathological phenotypes, such as necrotic fibres, phagocytosis, hypertrophy, splitting fibres, and fat accumulation (https://www.jax.org/strain/013149 (accessed on 1 August 2023)).

The **SJL mouse model** has a splice-site mutation conferring the in-frame deletion of 171 bp exon 45, corresponding to the fifth C2 domain of the protein. Its phenotype presents muscle weakness from 3 weeks of age and a more severe pathologic phenotype at 6 months [58] (https://www.jax.org/strain/000686 (accessed on 1 August 2023)). This mouse model has a 15% residual dysferlin expression [59].

Another mouse model contains the **human DYSF healthy transgene** (https://www.jax.org/strain/014146 (accessed on august 1st 2023)).

Some recent models contain a **point mutation** such as c.4079T>C (NM_001077694.1) in exon 38, which causes an amino acid change in the mouse sequence (p.L1360P) to recreate the human analogue c.4022T>C (p.Leu1341Pro) in the same exon [60]. Another model also contains a patient mutation c.3477C>A (NM_003494.3) in exon 32, which causes a stop codon instead of a tyrosine (p.Y1159X) [61].

## 5. Treatments

Though the last decades allowed researchers to evaluate many options, there is still no cure or efficient treatment approved for dysferlinopathy [34].

### 5.1. Symptomatic Treatments

The only treatments that are currently available for dysferlinopathy are used to treat the symptoms; none of them treat the root cause. Walking aids, physiotherapy, or occupational therapy are prescribed to improve patient quality of life. Ankle and foot orthoses are also used by some patients [62].

Some molecules have been tested in mice, such as ezetimibe, a cholesterol absorption blocker. Ezetimibe showed a significant reduction in fat accumulation in the triceps, gastrocnemius, and quadriceps muscles (84%, 78%, and 65%, respectively) and restored step length in dysferlin-deficient mice [63].

Another molecule, recombinant human galectin-1 (rHsGal-1), was tried in dysferlinopathy mouse models to increase the myogenic transcription factors involved in myotube formation and membrane repair [64]. The result was an increase in myotube formation for A/J −/− mice myotubes and an improved membrane repair in A/J −/− myotubes, and Dysf −/− and WT myofibers. It was also noticed that the improved repair is given by the carbohydrate recognition domain (CRD) of Gal-1.

### 5.2. Antisense-Mediated Exon Skipping

Antisense-mediated exon skipping uses antisense oligonucleotides (ASOs), a small DNA sequence, to skip one or more exons. Several possible modifications to the structure help the ASOs to resist against degradation and hybridize better with RNA [65]. A targeted region can be degraded by RNAse H, which recognizes the DNA (ASO)-RNA complex and cleaves the binding site to degrade its bound RNA. Another mechanism is to bind the targeted region to a site, creating a steric block against a start site, a splicing site, an RNA binding protein, or an upstream open-reading frame (uORF) to mask it during splicing or translation.

There are several ways to deliver ASOs, for example, by pairing them with the following: Triantennary N-acetylgalactosamine (GalNAc), peptides, lipids, antibodies or adaptamers, or to a stimuli-responsive structure. Other delivery methods include packaging the ASOs in a stable nucleic acid lipid particle, in exosomes, in spherical nucleic acid nanoparticles made with a gold core linked to ASOs with metal–thiol, or using a DNA cage with an ASO at its end [66].

In 2015, Barthélémy et al. showed that they could restore the plasma membrane resealing ability by skipping exon 32 in patient cells containing a stop codon in said exon [67]. Lee et al. reported restored human fibroblast plasma membrane resealing in vitro after skipping exons 26–27 or 28–29 [68]. Malcher et al. showed that exons 37 and 38 can also be skipped in MMex38 mice to restore resealing [69].

### 5.3. Myoblast Transplantation

Leriche-Guérin et al. (2002) showed that myoblast transplantation in SJL dysferlin-deficient mice resulted in the presence of 20 to 30% mouse dysferlin-positive fibres one month after the transplantation in the tibialis anterior muscle [70]. A parallel experiment in SCID mice transplanted with dysferlin-positive human cells resulted in 40 to 50% human dysferlin-positive fibres. This method uses intramuscular injections of dysferlin-positive cells from a donor with a correct dysferlin gene. Unfortunately, myoblasts do not migrate very far from their injection site; therefore, several injections per muscle are needed. Moreover, if the donor is not compatible with the receiver, it is necessary to immunosuppress the patient. A clinical trial in Canada for the same procedure to restore dystrophin in Duchenne Muscular Dystrophy (DMD) patients showed dystrophin in 34.5% of fibres in the grafted gastrocnemius 18 months post-transplantation [71] (NCT02196467).

### 5.4. Gene Editing

Gene editing started in the 1990s with I-SceI, which causes double-strand breaks (DSBs) and homologous recombination (HR) [72]. Other techniques also permit the induction of DSBs, such as zinc-finger nucleases (ZFNs) [73,74] and transcription activator-like effector nucleases (TALENs) [75].

The CRISPR system use a Cas9 nuclease and a single guide RNA (sgRNA) that binds to a sequence of 20 nucleotides to induce a DSB at a specific DNA site [76].

Base editing is derived from the basic CRISPR. It uses a Cas9 nickase fused with a cytidine deaminase to modify the cytidines in a narrow window of six nucleotides into a thymine. The Cas9 nickase may also be fused with an adenosine deaminase to change an adenosine into a guanine. Both modifications are performed without inducing a DSB [77,78], but they change all said bases in the window, not only one.

Prime editing uses a Cas9 nickase to cleave only one DNA strand. The Cas9 nickase is fused with a reverse transcriptase (RT) and uses a prime editing guide RNA (pegRNA) to edit a few nucleotides [79,80]. After one strand is cleaved by Cas9 nickase, the Primer Binding Site (PBS) of the pegRNA hybridizes with the free DNA strand. The reverse transcriptase then uses the reverse transcriptase template (RTT) of the pegRNA to synthesize a modified DNA strand.

However, these methods have their own limitations and still have to be tested. For example, double-strand breaks can cause unwanted insertions or deletions (indels) [81]. Another limit is that an efficient delivery method to the cells in vivo still has to be found. Long-term outcomes also have to be considered, such as the immune response to the viral vector, which cannot be used more than once [82].

In 2016, Turan et al. corrected the nonsense mutation c.5713C>T; p.R1905X in the *DYSF* gene in vitro [83]. They used single-stranded oligonucleotide-mediated gene editing along with CRISPR/Cas9 to increase the frequency of homology-directed repair. The dysferlin protein was rescued by their correction, as seen in a Western blot, but it was not quantified.

In 2019, Lek et al. designed prime editing constructions with the aim to correct mutations in the *DYSF* gene in vitro but needed a more efficient delivery method to enter the nuclei (https://www.jain-foundation.org/past-projects/the-use-of-crispr-as-a-potential-therapeutic-for-dysferlinopathy/ (accessed on 1 August 2023)).

Another approach is to deliver the dysferlin gene to the cells. Different truncated dysferlin molecules were designed to be contained in one AAV [84], and the full-length gene was successfully delivered in mice using two overlapping AAV vectors [85].

### 5.5. Clinical Trials

In 2003, a German study in phases 2 and 3 tested the use of Deflazacort in dysferlinopathy (NCT00527228). The trial was double-blind, including a placebo group, but the treatment showed no improvement in muscle strength and detrimental steroid side effects [86]. The conclusion of this study was that this steroid treatment is not appropriate for dysferlinopathy, which can be misdiagnosed as polymyositis, for which steroids can be used.

An American phase I clinical trial tested an intramuscular injection of rAAVrh.74.MHCK7.DYSF.DV in the extensor digitorum brevis muscle (EDB) from March 2016 to July 2019 (NCT02710500). The study was first conducted on mice and non-human primates and showed restored dysferlin expression in all muscle groups, as well as a restored function measured by membrane repair and diaphragm strength [87]. The clinical trial results have not yet been published.

Another clinical trial is evaluating the safety, efficacy, and tolerability of SRP-6004, a dual-vector AAV gene therapy, that is being administered intravenously in ambulatory patients (NCT05906251) [88].

## 6. Conclusions

In conclusion, dysferlinopathy has no efficient cure yet, but several researchers are studying potential treatments, and some of them have reached the clinical trial stage. The roles of dysferlin are being studied with the hope of identifying additional therapeutic pathways. The roles under investigation include membrane repair and vesicle fusion, T-tubule development and maintenance, Ca^2+^ signalling, lipid metabolism, and the regulation of various molecules. Recent mouse models that contain patient mutations have been created with the aim of evaluating the effects of different treatments, including genetic therapy. These research avenues involve not only symptomatic treatments but also treatments such as exon skipping, myoblast transplantation, and gene editing, which treat the root cause of dysferlinopathy by restoring dysferlin protein expression. The hope is that, in the near future, there will be treatments that will significantly improve the quality of life of those with dysferlinopathy.

## Figures and Tables

**Table 1:** Muscles involved in dysferlinopathy.

In the upper limb girdle: the subscapularis muscle is the most affected, and the levator scapulae is the least affected.
Paraspinal muscles ≥ abdominal muscles
Gluteus minimus ≥ gluteus medius and maximus
Obturator externus ≥ gluteus maximus
Adductor magnus ≥ adductor longus
Rectus femoris is also involved when the vasti muscles are involved
Peroneus ≥ tibialis anterior
Symmetric involvement of left and right sides

≥: is equally or more involved than.

## Data Availability

Not applicable.

## References

[1] Doherty K.R., Cave A., Davis D.B., Delmonte A.J., Posey A., Earley J.U., Hadhazy M., McNally E.M. (2005). Normal myoblast fusion requires myoferlin. Development.

[2] Matsumura T., Aoki M., Nagano A., Hayashi Y.K., Asada C., Ogawa M., Yamanaka G., Goto K., Nakagawa M., Oka H. (1999). Molecular genetic analysis of dysferlin in Japanese patients with Miyoshi myopathy. Proc. Jpn. Acad. Ser. B.

[3] Kerr J.P., Ziman A.P., Mueller A.L., Muriel J.M., Kleinhans-Welte E., Gumerson J.D., Vogel S.S., Ward C.W., Roche J.A., Bloch R.J. (2013). Dysferlin stabilizes stress-induced Ca^2+^ signaling in the transverse tubule membrane. Proc. Natl. Acad. Sci. USA.

[4] Liu J., Aoki M., Illa I., Wu C., Fardeau M., Angelini C., Serrano C., Urtizberea J.A., Hentati F., Ben Hamida M. (1998). Dysferlin, a novel skeletal muscle gene, is mutated in Miyoshi myopathy and limb girdle muscular dystrophy. Nat. Genet..

[5] Aoki M., Liu J., Richard I., Bashir R., Britton S., Keers S.M., Oeltjen J., Brown H.E.V., Marchand S., Bourg N. (2001). Genomic organization of the dysferlin gene and novel mutations in Miyoshi myopathy. Neurology.

[6] Nguyen K., Bassez G., Bernard R., Krahn M., Labelle V., Figarella-Branger D., Pouget J., Hammouda E.H., Béroud C., Urtizberea A. (2005). Dysferlin mutations in LGMD2B, Miyoshi myopathy, and atypical dysferlinopathies. Hum. Mutat..

[7] Matsuda C., Kameyama K., Tagawa K., Ogawa M., Suzuki A., Yamaji S., Okamoto H., Nishino I., Hayashi Y.K. (2005). Dysferlin interacts with affixin (β-parvin) at the sarcolemma. J. Neuropathol. Exp. Neurol..

[8] Ampong B.N., Imamura M., Matsumiya T., Yoshida M., Takeda S.I. (2005). Intracellular localization of dysferlin and its association with the dihydropyridine receptor. Acta Myol. Myopathies Cardiomyopathies Off. J. Medi-Terranean Soc. Myol..

[9] Huang Y., de Morrée A., van Remoortere A., Bushby K., Frants R.R., Dunnen J.T., van der Maarel S.M. (2008). Calpain 3 is a modulator of the dysferlin protein complex in skeletal muscle. Hum. Mol. Genet..

[10] Yan X., Kumar K., Lamarche R.M., Youssef H., Shaw G.S., Marcotte I., DeWolf C.E., Warschawski D.E., Boisselier E. (2021). Interactions between the Cell Membrane Repair Protein S100A10 and Phospholipid Monolayers and Bilayers. Langmuir.

[11] Lennon N.J., Kho A., Bacskai B.J., Perlmutter S.L., Hyman B.T., Brown R.H. (2003). Dysferlin Interacts with Annexins A1 and A2 and Mediates Sarcolemmal Wound-healing. J. Biol. Chem..

[12] Azakir B.A., Di Fulvio S., Therrien C., Sinnreich M. (2010). Dysferlin interacts with tubulin and microtubules in mouse skeletal muscle. PLoS ONE.

[13] Muriel J., Lukyanenko V., Kwiatkowski T., Bhattacharya S., Garman D., Weisleder N., Bloch R.J. (2022). The C2 domains of dysferlin: Roles in membrane localization, Ca^2+^ signalling and sarcolemmal repair. J. Physiol..

[14] Cárdenas A.M., González-Jamett A.M., Cea L.A., Bevilacqua J.A., Caviedes P. (2016). Dysferlin function in skeletal muscle: Possible pathological mechanisms and therapeutical targets in dysferlinopathies. Exp. Neurol..

[15] Cai C., Masumiya H., Weisleder N., Matsuda N., Nishi M., Hwang M., Ko J.K., Lin P., Thornton A., Zhao X. (2009). MG53 nucleates assembly of cell membrane repair machinery. Nat. Cell Biol..

[16] Wenzel K., Zabojszcza J., Carl M., Taubert S., Lass A., Harris C.L., Ho M., Schulz H., Hummel O., Hubner N. (2005). Increased susceptibility to complement attack due to down-regulation of decay-accelerating factor/CD55 in dysferlin-deficient muscular dystrophy. J. Immunol..

[17] Kobayashi K., Izawa T., Kuwamura M., Yamate J. (2012). Dysferlin and animal models for dysferlinopathy. J. Toxicol. Pathol..

[18] Davis D.B., Doherty K.R., Delmonte A.J., McNally E.M. (2002). Calcium-sensitive phospholipid binding properties of normal and mutant ferlin C2 domains. J. Biol. Chem..

[19] Bansal D., Campbell K.P. (2004). Dysferlin and the plasma membrane repair in muscular dystrophy. Trends Cell Biol..

[20] Hofhuis J., Bersch K., Büssenschütt R., Drzymalski M., Liebetanz D., Nikolaev V.O., Wagner S., Maier L.S., Gärtner J., Klinge L. (2017). Dysferlin mediates membrane tubulation and links T-tubule biogenesis to muscular dystrophy. J. Cell Sci..

[21] Therrien C., Di Fulvio S., Pickles S., Sinnreich M. (2009). Characterization of lipid binding specificities of dysferlin C2 domains reveals novel interactions with phosphoinositides. Biochemistry.

[22] Wang Y., Tadayon R., Santamaria L., Mercier P., Forristal C.J., Shaw G.S. (2021). Calcium binds and rigidifies the dysferlin C2A domain in a tightly coupled manner. Biochem. J..

[23] Nagaraju K., Rawat R., Veszelovszky E., Thapliyal R., Kesari A., Sparks S., Raben N., Plotz P., Hoffman E.P. (2008). Dysferlin deficiency enhances monocyte phagocytosis: A model for the inflammatory onset of limb-girdle muscular dystrophy 2B. Am. J. Pathol..

[24] Lostal W., Bartoli M., Roudaut C., Bourg N., Krahn M., Pryadkina M., Borel P., Suel L., Roche J.A., Stockholm D. (2012). Lack of correlation between outcomes of membrane repair assay and correction of dystrophic changes in experimental therapeutic strategy in dysferli-nopathy. PLoS ONE.

[25] ClinVar. https://www.ncbi.nlm.nih.gov/clinvar/?term=dysf%5Bgene%5D&redir=gene.

[26] Straub V., Murphy A., Udd B., Corrado A., Aymé S., Bönneman C., de Visser M., Hamosh A., Jacobs L., Khizanishvili N. (2018). 229th ENMC international workshop: Limb girdle muscular dystrophies–Nomenclature and reformed classification Naarden, The Netherlands, 17–19 March 2017. Neuromuscul. Disord..

[27] Patel N.J., Van Dyke K.W., Espinoza L.R. (2017). Limb-girdle muscular dystrophy 2B and miyoshi presentations of dysferlinopathy. Am. J. Med. Sci..

[28] Illarioshkin S.N., Ivanova–Smolenskaya I.A., Greenberg C.R., Nylen E., Sukhorukov V.S., Poleshchuk V.V., Markova E.D., Wroge-mann K. (2000). Identical dysferlin mutation in limb-girdle muscular dystrophy type 2B and distal myopathy. Neurology.

[29] Weiler T., Bashir R., Anderson L.V.B., Davison K., Moss J.A., Britton S., Nylen E., Keers S., Vafiadaki E., Greenberg C.R. (1999). Identical mutation in patients with limb girdle muscular dystrophy type 2B or miyoshi myopathy suggests a role for modifier gene(s). Hum. Mol. Genet..

[30] Moore U., Gordish H., Diaz-Manera J., James M.K., Mayhew A.G., Guglieri M., Fernandez-Torron R., Rufibach L.E., Feng J., Blamire A.M. (2021). Miyoshi myopathy and limb girdle muscular dystrophy R2 are the same disease. Neuromuscul. Disord..

[31] Woudt L., Di Capua G.A., Krahn M., Castiglioni C., Hughes R., Campero M., Trangulao A., González-Hormazábal P., Godoy-Herrera R., Lévy N. (2015). Toward an objective measure of functional disability in dysferlinopathy. Muscle Nerve.

[32] Harris E., Bladen C.L., Mayhew A., James M., Bettinson K., Moore U., Smith F.E., Rufibach L., Cnaan A., Bharucha-Goebel D.X. (2016). The Clinical Outcome Study for dysferlinopathy: An international multicenter study. Neurol. Genet..

[33] Aoki M., Takahashi T. (2005). Mutational and clinical features of Japanese patients with dysferlinopathy (Miyoshi myopathy and limb girdle muscular dystrophy type 2B). Rinsho Shinkeigaku.

[34] Socoliuc C.G., Oprişan A., Dobrescu A., Manole E., Bastian A.E. (2021). The challenging diagnosis of dysferlinopathy. Rom. J. Neurol..

[35] Moore U., Fernandez-Torron R., Jacobs M., Gordish-Dressman H., Diaz-Manera J., James M.K., Mayhew A.G., Harris E., Guglieri M., Rufibach L.E. (2022). Cardiac and pulmonary findings in dysferlinopathy: A 3-year, longitudinal study. Muscle Nerve.

[36] Llansó L., Moore U., Bolano-Diaz C., James M., Blamire A.M., Carlier P.G., Rufibach L., Gordish-Dressman H., Boyle G., Hilsden H. (2023). Expanding the muscle imaging spectrum in dysferlinopathy: Description of an outlier population from the classical MRI pattern. Neuromuscul. Disord..

[37] Reyngoudt H., Smith F.E., Araújo E.C.d.A., Wilson I., Fernández-Torrón R., James M.K., Moore U.R., Díaz-Manera J., Marty B., Azzabou N. (2022). Three-year quantitative magnetic resonance imaging and phosphorus magnetic resonance spectroscopy study in lower limb muscle in dysferlinopathy. J. Cachexia Sarcopenia Muscle.

[38] Diaz-Manera J., Fernandez-Torron R., Llauger J., James M.K., Mayhew A., Smith F.E., Moore U.R., Blamire A.M., Carlier P.G., Rufibach L. (2018). Muscle MRI in patients with dysferlinopathy: Pattern recognition and implications for clinical trials. J. Neurol. Neurosurg. Psychiatry.

[39] Rekik S., Sakka S., Ben Romdhane S., Amer Y.B., Lehkim L., Farhat N., Ben Mahfoudh K., Authier F.J., Dammak M., Mhiri C. (2020). Novel splicing dysferlin mutation causing myopathy with intra-familial heterogeneity. Mol. Biol. Rep..

[40] Selcen D., Stilling G., Engel A.G. (2001). The earliest pathologic alterations in dysferlinopathy. Neurology.

[41] Spuler S., Engel A.G. (1998). Unexpected sarcolemmal complement membrane attack complex deposits on nonnecrotic muscle fibers in muscular dystrophies. Neurology.

[42] Fernández-Eulate G., Querin G., Moore U., Behin A., Masingue M., Bassez G., Leonard-Louis S., Laforêt P., Maisonobe T., Merle P. (2021). Deep phenotyping of an international series of patients with late-onset dysferlinopathy. Eur. J. Neurol..

[43] Folland C., Johnsen R., Gomez A.B., Trajanoski D., Davis M.R., Moore U., Straub V., Barresi R., Guglieri M., Hayhurst H. (2022). Identification of a novel heterozygous *DYSF* variant in a large family with a dominantly-inherited dysferlinopathy. Neuropathol. Appl. Neurobiol..

[44] Eryaşar G., Seçil Y., Beckmann Y., Kendir A.İ., Diniz A.G., Başoğlu M. (2011). Two cases with dysferlinopathy. Turk. Norol. Derg..

[45] de Luna N., Gallardo E., Illa I. (2004). In vivo and in vitro dysferlin expression in human muscle satellite cells. J. Neuropathol. Exp. Neurol..

[46] Contreras-Cubas C., Barajas-Olmos F., Frayre-Martínez M.I., Siordia-Reyes G., Guízar-Sánchez C.C., García-Ortiz H., Orozco L., Baca V. (2022). Dysferlinopathy misdiagnosed with juvenile polymyositis in the pre-symptomatic stage of hyperCKemia: A case report and literature review. BMC Med. Genom..

[47] Urtizberea J.A., Bassez G., Leturcq F., Nguyen K., Krahn M., Levy N. (2008). Dysferlinopathies. Neurol. India.

[48] Fanin M., Angelini C. (2016). Progress and challenges in diagnosis of dysferlinopathy. Muscle Nerve.

[49] Ho M., Gallardo E., McKenna-Yasek D., De Luna N., Illa I., Brown R.H. (2002). A novel, blood-based diagnostic assay for limb girdle muscular dystrophy 2B and Miyoshi myopathy. Ann. Neurol. Off. J. Am. Neu-Rological Assoc. Child Neurol. Soc..

[50] Ankala A., Nallamilli B.R., Rufibach L.E., Hwang E., Hegde M.R. (2014). Diagnostic overview of blood-based dysferlin protein assay for dysferlinopathies. Muscle Nerve.

[51] Anderson L.V., Harrison R.M., Pogue R., Vafiadaki E., Pollitt C., Davison K., Moss J.A., Keers S., Pyle A., Shaw P.J. (2000). Secondary reduction in calpain 3 expression in patients with limb girdle muscular dystrophy type 2B and Miyoshi myopathy (primary dys-ferlinopathies). Neuromuscul. Disord..

[52] Sinclair A. (2002). Genetics 101: Detecting mutations in human genes. Can. Med. Assoc. J..

[53] Moore U., Jacobs M., James M.K., Mayhew A.G., Fernandez-Torron R., Feng J., Cnaan A., Eagle M., Bettinson K., Rufibach L.E. (2019). Assessment of disease progression in dysferlinopathy: A 1-year cohort study. Neurology.

[54] Mayhew A.G., James M.K., Moore U., Sutherland H., Jacobs M., Feng J., Lowes L.P., Alfano L.N., Lofra R.M., Rufibach L.E. (2022). Assessing the Relationship of Patient Reported Outcome Measures with Functional Status in Dysferlinopathy: A Rasch Analysis Approach. Front. Neurol..

[55] Lostal W., Bartoli M., Bourg N., Roudaut C., Bentaïb A., Miyake K., Guerchet N., Fougerousse F., McNeil P., Richard I. (2010). Efficient recovery of dysferlin deficiency by dual adeno-associated vector-mediated gene transfer. Hum. Mol. Genet..

[56] Nagy N., Nonneman R.J., Llanga T., Dial C.F., Riddick N.V., Hampton T., Moy S.S., Lehtimäki K.K., Ahtoniemi T., Puoliväli J. (2017). Hip region muscular dystrophy and emergence of motor deficits in dysferlin-deficient Bla/J mice. Physiol. Rep..

[57] Ho M., Post C.M., Donahue L.R., Lidov H.G., Bronson R.T., Goolsby H., Watkins S.C., Cox G.A., Brown R.H. (2004). Disruption of muscle membrane and phenotype divergence in two novel mouse models of dysferlin deficiency. Hum. Mol. Genet..

[58] Weller A.H., Magliato S.A., Bell K.P., Rosenberg N.L. (1997). Spontaneous myopathy in the SJL/J mouse: Pathology and strength loss. Muscle Nerve Off. J. Am. Assoc. Electrodiagn. Med..

[59] Bittner R.E., Anderson L.V., Burkhardt E., Bashir R., Vafiadaki E., Ivanova S., Raffelsberger T., Maerk I., Höger H., Jung M. (1999). Dysferlin deletion in SJL mice (SJL-Dysf) defines a natural model for limb girdle muscular dystrophy 2B. Nat. Genet..

[60] Heidt L., Bader M., Spuler S., Schoewel V. (2014). GP 284: Dysferlinopathy caused by protein misfolding: The novel murine animal model Dysf-MMex38. Neuromuscul. Disord..

[61] Ballouhey O., Chapoton M., Alary B., Courrier S., Da Silva N., Krahn M., Lévy N., Weisleder N., Bartoli M. (2023). A Dysferlin Exon 32 Nonsense Mutant Mouse Model Shows Pathological Signs of Dysferlinopathy. Biomedicines.

[62] Straub V., Bushby K. (2008). Therapeutic possibilities in the autosomal recessive limb-girdle muscular dystrophies. Neurotherapeutics.

[63] White Z., Theret M., Milad N., Tung L.W., Chen W.W., Sirois M.G., Rossi F., Bernatchez P. (2021). Cholesterol absorption blocker ezetimibe prevents muscle wasting in severe dysferlin-deficient and *mdx* mice. J. Cachexia Sarcopenia Muscle.

[64] Vallecillo-Zúniga M.L., Rathgeber M.F., Poulson P.D., Hayes S., Luddington J.S., Gill H.N., Teynor M., Kartchner B.C., Valdoz J., Stowell C. (2020). Treatment with galectin-1 improves myogenic potential and membrane repair in dysferlin-deficient models. PLoS ONE.

[65] Rinaldi C., Wood M.J.A. (2018). Antisense oligonucleotides: The next frontier for treatment of neurological disorders. Nat. Rev. Neurol..

[66] Roberts T.C., Langer R., Wood M.J.A. (2021). Advances in oligonucleotide drug delivery. Nat. Rev. Drug Discov..

[67] Barthélémy F., Blouin C., Wein N., Mouly V., Courrier S., Dionnet E., Kergourlay V., Mathieu Y., Garcia L., Butler-Browne G. (2015). Exon 32 skipping of dysferlin rescues membrane repair in patients’ cells. J. Neuromuscul. Dis..

[68] Lee J.J., Maruyama R., Duddy W., Sakurai H., Yokota T. (2018). Identification of novel antisense-mediated exon skipping targets in DYSF for therapeutic treatment of dysferlinopathy. Mol. Ther. Nucleic Acids.

[69] Malcher J., Heidt L., Goyenvalle A., Escobar H., Marg A., Beley C., Benchaouir R., Bader M., Spuler S., García L. (2018). Exon skipping in a Dysf-missense mutant mouse model. Mol. Ther. Nucleic Acids.

[70] Leriche-Guérin K., Anderson L., Wrogemann K., Roy B., Goulet M., Tremblay J. (2002). Dysferlin expression after normal myoblast transplantation in SCID and in SJL mice. Neuromuscul. Disord..

[71] Skuk D., Goulet M., Roy B., Piette V., Côté C.H., Chapdelaine P., Hogrel J.-Y., Paradis M., Bouchard J.-P., Sylvain M. (2007). First test of a “high-density injection” protocol for myogenic cell transplantation throughout large volumes of muscles in a Duchenne muscular dystrophy patient: Eighteen months follow-up. Neuromuscul. Disord..

[72] Jasin M. (1996). Genetic manipulation of genomes with rare-cutting endonucleases. Trends Genet..

[73] Durai S., Mani M., Kandavelou K., Wu J., Porteus M.H., Chandrasegaran S. (2005). Zinc finger nucleases: Custom-designed molecular scissors for genome engineering of plant and mammalian cells. Nucleic Acids Res..

[74] Carroll D. (2008). Zinc-finger Nucleases as Gene Therapy Agents. Gene Ther..

[75] Sun N., Zhao H. (2013). Transcription activator-like effector nucleases (TALENs): A highly efficient and versatile tool for genome editing. Biotechnol. Bioeng..

[76] Hwang W.Y., Fu Y., Reyon D., Maeder M.L., Tsai S.Q., Sander J.D., Peterson R.T., Yeh J.-R.J., Joung J.K. (2013). Efficient genome editing in zebrafish using a CRISPR-Cas system. Nat. Biotechnol..

[77] Gaudelli N.M., Komor A.C., Rees H.A., Packer M.S., Badran A.H., Bryson D.I., Liu D.R. (2017). Programmable base editing of A•T to G•C in genomic DNA without DNA cleavage. Nature.

[78] Porto E.M., Komor A.C., Slaymaker I.M., Yeo G.W. (2020). Base editing: Advances and therapeutic opportunities. Nat. Rev. Drug Discov..

[79] Godbout K., Tremblay J.P. (2023). Prime editing for human gene therapy: Where are we now?. Cells.

[80] Anzalone A.V., Randolph P.B., Davis J.R., Sousa A.A., Koblan L.W., Levy J.M., Chen P.J., Wilson C., Newby G.A., Raguram A. (2019). Search-and-replace genome editing without double-strand breaks or donor DNA. Nature.

[81] Cisneros-Aguirre M., Ping X., Stark J.M. (2022). To indel or not to indel: Factors influencing mutagenesis during chromosomal break end joining. DNA Repair.

[82] Calcedo R., Wilson J.M. (2013). Humoral Immune Response to AAV. Front. Immunol..

[83] Turan S., Farruggio A.P., Srifa W., Day J.W., Calos M.P. (2016). Precise correction of disease mutations in induced pluripotent stem cells derived from patients with limb girdle muscular dystrophy. Mol. Ther..

[84] Azakir B.A., Di Fulvio S., Salomon S., Brockhoff M., Therrien C., Sinnreich M. (2012). Modular dispensability of dysferlin C2 domains reveals rational design for mini-dysferlin molecules. J. Biol. Chem..

[85] Pryadkina M., Lostal W., Bourg N., Charton K., Roudaut C., Hirsch M.L., Richard I. (2015). A comparison of AAV strategies distinguishes overlapping vectors for efficient systemic delivery of the 6.2 kb Dysferlin coding sequence. Mol. Ther. Methods Clin. Dev..

[86] Walter M.C., Reilich P., Thiele S., Schessl J., Schreiber H., Reiners K., Kress W., Müller-Reible C., Vorgerd M., Urban P. (2013). Treatment of dysferli-nopathy with deflazacort: A double-blind, placebo-controlled clinical trial. Orphanet J. Rare Dis..

[87] Sondergaard P.C., Griffin D.A., Pozsgai E.R., Johnson R.W., Grose W.E., Heller K.N., Shontz K.M., Montgomery C.L., Liu J., Clark K.R. (2015). AAV. dysferlin overlap vectors restore function in dysferlinopathy animal models. Ann. Clin. Transl. Neurol..

[88] Clinical Trials, Study NCT05906251. NCT05906251.

